# Identification of a Novel *KCNQ1* Frameshift Mutation and Review of the Literature among Iranian Long QT Families

**DOI:** 10.29252/.23.3.228

**Published:** 2019-05

**Authors:** Azam Amirian, Zahra Zafari, Morteza Karimipoor, Alireza Kordafshari, Mohammad Dalili, Siamak Saber, Amir Farjam Fazelifar, Sirous Zeinali

**Affiliations:** 1 Department of Molecular Medicine, Biotechnology Research Center, Pasteur Institute of Iran, Tehran, Iran; 2 Department of Biology, Shahed University, Tehran, Iran; 3 Cardiac Electrophysiology Research Center, Rajaie Cardiovascular Medical, and Research Center, Iran University of Medical Sciences, Tehran, Iran; 4 Medical Genetics Laboratory, Kawsar Human Genetics Research Center, No. 41 Majlesi St., Vali Asr St., Tehran, Iran

**Keywords:** Jervell-Lange-Nielsen syndrome, *KCNQ1*, Long QT syndrome, Romano-Ward syndrome

## Abstract

**Background:**

Long QT syndrome (LQTS) is characterized by the prolongation of QT interval, which results in syncope and sudden cardiac death in young people. *KCNQ1* is the most common gene responsible for this syndrome.

**Methods:**

Molecular investigation was performed by DNA Sanger sequencing in Iranian families with a history of syncope. *In silico* examinations were performed for predicting the pathogenicity of the novel variant.

**Results:**

A novel homozygous *KCNQ1* frameshift mutation, c.1426_1429delATGC (M476Pfs*4), was identified, and then the current literatures of five patients were reviewed regarding the LQTS.

**Conclusion:**

The novel frameshift mutation has been reported for the first time among the Iranian population. Our finding along with the case series study of LQTS patients illustrates the importance of genetic and case series in precise detection of the frequency of LQTS carriers.

## INTRODUCTION

Long-QT syndrome (LQTS) is characterized by prolonged QT interval on the electro-cardiograms (ECG), syncope, and cardiac arrest[1,2]. It has been reported that mutations in 16 genes can result in inherited LQTS[3]. There are two main types of inherited LQTS: the autosomal dominant termed Romano-Ward syndrome (RWS) and the autosomal recessive type with less recurrence defined as Jervell-Lange Nielsen Syndrome (JLNS)[3]. Mutations in *KCNQ1* gene may create both the RWS and JLNS disorders.

JLNS is a condition in which QTc prolongation accompanying with congenital two-sided deafness occurs due to compound heterozygous or homozygous mutations in *KCNQ1* and/or *KCNE1* genes[4]. RWS is recognized as the aggregation of symptoms such as syncopal attacks and QT interval alteration in ECG without congenital deafness[5]. Although RWS is mostly inherited in an autosomal dominant pattern[6], autosomal recessive inheritance is associated with homozygosity[7]. In addition, the association of JLNS with compound heterozygosity[8] has been observed in some cases; the fact indicates that the pattern of inheritance for this disease could be more intricate.

In this study, we present a novel homozygous frameshift mutation of *KCNQ1* gene and review recent literatures on novel frameshift and recessive *KCNQ1* mutations of Iranian families.

## MATERIALS AND METHODS

### Clinical evaluation

Six unrelated LQTS patients (patients A-F) were referred to Emergency Department of Rajaie Cardiovascular, Medical and Research Center (Tehran, Iran) for further clinical evaluations and molecular testing.

### Genetic study

Informed consent and the study approval by the Ethics Committee of Pasteur Institute of Iran and Rajaie Cardiovascular, Medical and Research Center (adopted from the 1975 Helsinki Declaration) were obtained. Blood samples along with the ECG were collected from the patient and family members. Using the standard salting-out protocol, genomic DNA was extracted from the peripheral blood samples. Primer design was performed by Gene Runner and Primer3 online software for coding exons and exon-intron boundaries and also untranslated regions of *KCNQ1* (NM_000218). Primers for *KCNH2* (NM_000238) and *SCN5A* (NM_198056.2) were designed as well (Table 1). By PCR, exons of the genes were amplified, and by Sanger sequencing technique, the amplicons were sequenced. Sanger sequencing results were compared with the RefSeq genomic accession numbers, and the variants were evaluated by Mutation Database. For predicting the functional impact of the variant on the protein, MutationTaster[9], HSF[10], Mutation Assessor[11], SIFT[12], PolyPhen-2[13], and FATHMM[14] were used.

**Table 1:** Primers of *KCNQ1*, *KCNH2*, and *SCN5A* genes for coding regions

Gene/exon	Primer sequence	Gene/exon	Primer sequence
*KCNQ1* /1	F: AGCGGGATAGATGACACGAG	*SCN5A* /2	F: CCTCTCTGCAAATGGTGTCC
	R: CTTCCTGAGAGCTGGTGTGG		R: GGAAATGAGTCACTGGTGATCT
*KCNQ1* /2	F: TACCAGCTAATGGATGACTGG	*SCN5A* /3	F: CTGACCTGCCAAATGTGCTG
	R: GGTGACTCTGTTCCTGGGTTA		R: CCTAAGACAAATGCATGGTCAT
*KCNQ1* /3	F: TGGACATGAGCTGAAGCTGC	*SCN5A* /4	F: TGCCTATTAGGTGTCATGGAG
	R: ACACCATGATCAGCGTCTGAG		R: CTTCTGGCATTAATTTGAGTTG
*KCNQ1* /4	F: GTCTCTCCGTTTAGATGCTGC	*SCN5A* /5	F: TCGTTAGCCAGATGTTTAGAGC
	R: GGAATCTGGAGGTACCTGGC		R: CAGTCCACATGCAGCTCTGC
*KCNQ1* /5	F: CTGTCGGGATGGACATATACC	*SCN5A* /6	F: TCAGTTATCCCAGGTAAGATGC
	R: CCACACTAGGACAGCTTGAGAT		R: GGCTATTGGCAGTGGACATG
*KCNQ1* /6	F: ACCGGAGTTGTGAGGAGTGG	*SCN5A /*7	F: GAAATCAGGACAGAATCTCAGC
	R: CCAAAGGACTCAACACTGAGC		R: AGGACAGACGGGTAGCAGAC
*KCNQ1* /7	F: TTACATGTGCTGGTGGGACA	*SCN5A /*8	F: TCCCGTGTCTTCTGAGAGCA
	R: CTGGAGTATAGCACCTTCTAGAAG		R: GCACAGAGGAGACAGCTTCT
*KCNQ1* /8	F: CTTCCAGCACTGACCATACCT	*SCN5A /*9	F: ACAGCACGAACAAAGTCACG
	R: GCATTGGAGCCTGTCTTCCTC		R: AGGATGCTCTCTGCTCTGTGA
*KCNQ1* /9	F: CCACCTTTGCAAGTCTCTCC	*SCN5A /*10	F: CTCTGCAGGTCAGTACATGTCC
	R: CGATGCTAGGTTCCTGCCATC		R: GTGAATGTGGTATCGCTGAGTA
*KCNQ1* /10	F: CTGTGTGAAGACACTGGAGCTG	*SCN5A /*11	F: CTGTCTGAGTTTATCTCCATGATG
	R: GGTCTCTGACAACGAGGTATGAA		R: CCATAAGAGTGAGGGTCCATT
*KCNQ1* /11	F: TGATTGTCAGTGCTGGAGCT	*SCN5A /*12	F: CAAGCCCAGTTAAGTTTCAGG
	R: GTGCTATCTACTCGCCTAGTGC		R: CTCTAGGTGCATAAACTTACA
*KCNQ1* /12	F: GGACATGGCCTAAGTATCTCC	*SCN5A /*13	F: TGTCCCATCAAGACCTTCATC
	R: CCTATCTGAGACCTGACAGTGC		R: CTGTTCTGTGTAGCCTTGCC
*KCNQ1* /13	F: CGGTGAGTAGACAGGAAGCTG	*SCN5A /*14	F: TGTCCTGATAATCTCTCCTGTCC
	R: GAGTTCTTGCCTCTCAACCAC		R: GCTGAGAAATGTAGATTTGGAGT
*KCNQ1* /14	F: AACTAGCTCCGTGTGTTACAGG	*SCN5A /*15	F: TGCCTGGTATGCTTGGTGAG
	R: TGCATGACATGAAATGAAAGC		5´-GTCATGCCTTCACCCAACAG
*KCNQ1* /15	F: TCAGAGGTGGAGAGCGTGGA	*SCN5A /*16	F: GAAACAGTAGTGGGTGCTCTGG
	R: CGTAGTCTGCTTTGTGCTCTG		R: CACCAATGAACACACCAATCTAT
*KCNQ1* /16	F: AGACATAGGGTGCACACGTG	*SCN5A /*17	F: AAGCCTCGGAGCTGTTTGTC
	R: CGTCTCAGGTCTGAGTTGTTAC		R: CTCCCTTCCTAACTCAGTCCAG
*KCNH2 /*1	F: GCCACCCGAAGCCTAGTGC	*SCN5A /*18	F: GAGGAGTCTTCAGTGAGATGGAG
	R: GGAAACTCAGCTCAGGCTTTTGG		R: CTCTGATGCAGGACTAACCCA
*KCNH2 /*2	F: CTGTGTGAGTGGAGAATGTGG	*SCN5A /*19	F: AGCCTTAGACTCCAGCAGACC
	R: GGAGTTGCTAGGCTGTGGGT		R: TCCATCTGCCTGACGTGTCT
*KCNH2 /*3	F: GCAGAAGAAAGGATCATAGCC	*SCN5A /*20	F: CCATCCTCCTCAAAGAGTGC
	R: CCAGAATCACAGGTCCTTGG		R: GGAACAGCTCACAAACTCTCAA
*KCNH2 /*4	F: TGAAGTGTCACTTCAGATATGG	*SCN5A /*21	F: CTCATCTAGTTCCTGTTTCTGCT
	R: GTCCATTCATCCCATTACATT		R: CGTAAGTCTGAGTGACCCAGG
*KCNH2 /*5	F: TGGCTGCTTCCTTAGAGTGG	*SCN5A /*22	F: CCAGAAGCCAGGATACTCTTG
	R: GCAATCTATTCCAGAGCTGC		R: AGGCTAGGCAGCTGTGAGAA
*KCNH2 /*6	F: GTGGGCATTCTGATGGAAGCT	*SCN5A /*23	F: GGAAACCAGATGTTCTGAAGC
	R: CCTATGCTCCTTCTCTCCACA		R: CAGTTCTAGAACCGATACCATGT
*KCNH2 /*7	F: AGGAGGAGGGTCTAGGAAGTC	*SCN5A /*24	F: CTGACCACCCAGGCATTTAG
	R: TCGACGCTGAGACTGAGACA		R: GTCACTCTGGAGTTCAGCCTC
*KCNH2 /*8	F: TGGAGCGCAGATGTACAAGG	*SCN5A /*25	F: TAGTGACCTTCCTCTAGATACACC
	R: CACAGTCAGTAGTAAGGACCCTG		R: CCTGTAAGAACGTAAGAAGGG
*KCNH2 /*9	F: CTGATGCTTCCGAGATCTCC	*SCN5A /*26	F: GGTGGATACTGGATTTGCAG
	R: GAGGAAGAAATGCTAGCCTGG		R: AGGTATGATAAGGATGTAGCATC
*KCNH2 /*10	F: TGCAGTGATTGGCTAAGAGG	*SCN5A /*27	F: GGACAGCCAGTGGCTTTAGC
	R: TGGTATCATAGAGCAGCCTACA		R: CTGAATGCCATGTACAACCCT
*KCNH2 /*11	F: TCAAATGGTATCATAGAGCAGC	*SCN5A /*28.1	F: GCTCCTTGCCATATAGAGACC
	R: CCTGAAGCACCATTGCCAGT		R: CATCGTGGTCAACATGTACATT
*KCNH2 /12,*13	F: CAAGATAGCAGAAGAAGCGAC	*SCN5A /*28.2	F: GACATGTTCAACTTCCAGACCT
	R: AGCTGGATCCCCTTCTTCCA		R: GGTTATCCAGAGAGCCTTCC
*KCNH2 /*14	F: GGTGTTGTCTGGTAGAGGGAG	*SCN5A /*28.3	F: CCCAACCAGATAAGCCTCAT
	R: GTGGTCTCCAAGAACTGACTGA		R: GGAGTAAGAAATGGGCCTCA
*KCNH2 /*15	F: GCTCCTGCTCTCAGAGAATGC	-	-
	R: GGAACTCGAAAGCACAGCTC		

## RESULTS

**Fig 1:**
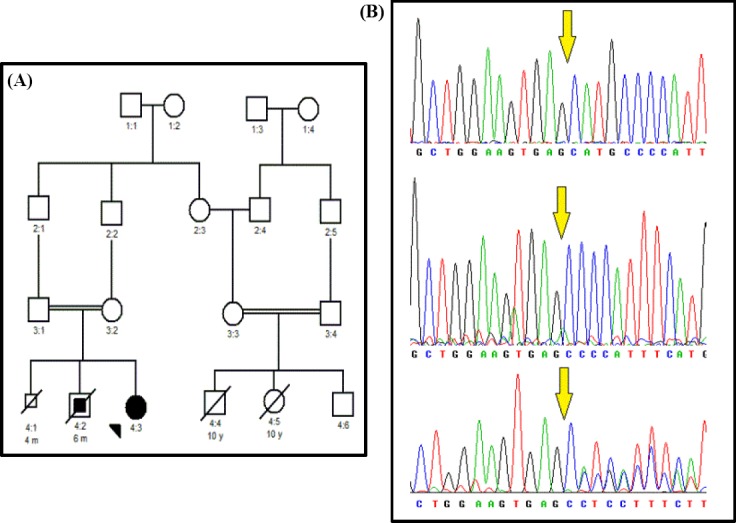
Pedigree and mutation confirmation. (A) Family pedigree for the patient A; (B) DNA Sanger sequencing confirmation of c.1426_1429delATGC (M476Pfs*4) mutation (arrows) in the index case with homozygote condition (middle), unaffected heterozygote father (lower), and a normal control sequence (upper).

**Fig 2:**
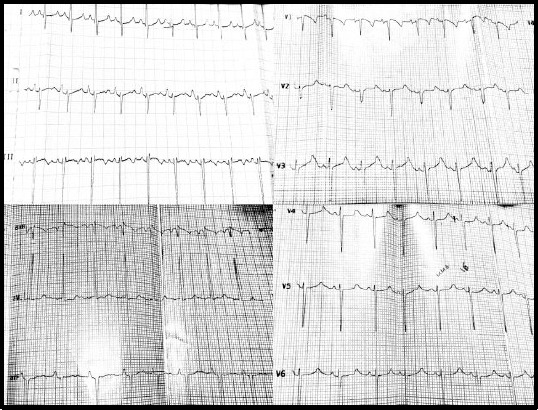
The electrocardiogram (ECG) of the index case. The 12-lead ECG of the patient A at five years of age (QTc over 500 ms in lead II; heart rate, 72 beat/min; with a paper speed of 25 mm/s and 10 mm/mV at 20 Hz).

Patient A was a five-year-old girl with a history of syncope and seizure disorders. She was offspring of a consanguineous marriage and suffered from congenital sensorineural deafness. She had experienced fainting around the age of 2.5. There was no family history of convulsions, but a sudden cardiac death happened in proband’s sibling at six months of age (4:2 in Fig. 1). One stillbirth was reported by her mother in the previous pregnancy. Her first ECG demonstrated normal sinus rhythm with the prolonged QT interval of over 500 miliseconds (ms), as depicted in Figure. 2, while her parents showed normal ECG.

Data of Sanger sequencing demonstrated a novel homozygous frameshift mutation, c.1426_1429 delATGC (M476Pfs*4; ClinVar accession number: SCV000678249.1), in the *KCNQ1* gene (Fig. 1A). The mutation was confirmed in the father of proband in heterozygous form, but the DNA sample of her mother was not available. The variant was considered as a frameshift mutation according to MutationTaster software. All the aforementioned predictive tools predicted it as a damaging and disease-causing variant.

This mutation introduces three novel amino acids after codon 475 (methionine 476, as the first affected amino acid, was changed to proline) and premature stop codon at 480 that resulted in a truncated protein.

Patients B and C were referred to the clinic due to syncope and a prolonged QTs interval of >600 and 560 ms, respectively. Except for hearing defect, physical and neurological examinations were totally normal, and also there was no electrolyte imbalance. Sanger sequencing of the *KCNQ1* gene for both cases showed a homozygous frameshift mutation, c.733_734delGG (p.G245Rfs*39), in the exon 5. This frameshift results in a premature stop codon and develops a truncated protein. Examination of the parents of two patients showed heterozygosity for this mutation. In our previous study[15], using STR markers, we demonstrated this frameshift mutation in two obviously unrelated families with the same origin, which may represent a founding effect.

Patients D and E were presented by the recurrent episodes of syncope and a prolonged QTs interval of >550 and 600 ms, respectively. Analysis of the DNA sequence in patient D revealed a homozygous missense mutation of c.1691A>G in the *KCNQ1* gene. The same mutation was also identified in patient E by next-generation sequencing. This variant has not previously been reported in Exome Aggregation Consortium (ExAC) or 1000 Genome databases. Co-segregation analysis confirmed the association of LQTS with the mentioned variant, and a same haplotype was detected in the two families. Using *in silico* analysis, we identified the p.D564G variant as a deleterious variant.

Patient F had a background of recurrent syncope, which the first episode occurred at the age of 18 months. Resting 12-lead ECG showed noticeably a prolonged QTc interval of >580 ms. Echocardiography detected a structurally normal heart, and the ECG of her parents was normal. Sanger sequencing of the *KCNQ1* gene exhibited a homozygous mutation, c.1532_1534delG (p. A512Pfs*81), that led to a premature stop codon. Her parents were heterozygous for this mutation. This variant has not been reported in the literature and databases before.

## DISCUSSION

In the present research, the mutation analysis of the *KCNQ1*, *KCNH2*, and *SCN5A* genes were performed in a patient among a cohort of 30 unrelated Iranian LQTS families. The sequence analysis of the index patient demonstrated the absence of mutation in the *SCN5A* and *KCNH2* genes but showed a novel homozygous mutation, c.1426_1429delATGC (M476Pfs*4), in *KCNQ1* gene. Five patients screened previously were found to have a missense mutation in two RWS families[16] and two frameshift mutations in three JLNS families[17,18].

The novel mutation was detected neither in ExAC nor in 1000 Genome; additionally, it has not been reported in any disease database such as HGMD and LOVD. In the index case, the 4-bp deletion was located at positions 1426 to 1429 in exon 11 of *KCNQ1* gene, which led to amino acid sequence alteration and was accordingly considered as a frameshift variant.

It has been shown that frameshift mutations leading to truncated proteins are responsible for the majority of JLNS cases[19,20]. Wei *et al*.[21] have reported a one-nucleotide deletion in the position 1188 of *KCNQ1* gene, which causes a frameshift and leads to a premature stop codon and consequently, results in a 259-amino-acid deletion in the C-terminal. Another study has demonstrated that a frameshift may lead to the loss of function of the potassium channel, due to a single nucleotide insertion onto the position 1149 of this gene[22]. In LQTS patients, frameshift mutation has been predicted to be a pathogenic mutation with an estimated predicted value of 99%[23]. In concordance with the genetic study result, our patient showed the clinical features of seizure, recurrent episodes of syncope, and the family history of serious heart events such as sudden cardiac death in the sibling of the proband who died at six months of age (4:2 in Fig. 1). Likewise, a congenital sensorineural deafness was observed in the proband in agreement with most previous reports in JLNS patients[24,25].

The probands B, C, and F were categorized as patients of JLNS, the recessive form of LQTS. A frameshift mutation (p. A512Pfs*81) was found in patient F. In B and C non-consanguineous families, the frameshift mutation, c.733_734delGG, happened in the C-loop between the transmembrane domains S4-S5 of the protein. The C-loop domains have a significant role in changing the potassium channel’s function; therefore, mutations in the residues of this region may impair the voltage-dependent activation of the channel, hence leading to increased risk of lethal cardiac events[26]. Functional assay showed that mutations in the C-loop of *KCNQ1* gene impress the adrenergic regulation of channel, either in the absence or in the presence of wild-type subunits[27,28].

To confirm whether the novel missense variant of c.1691A>G (p.D564G) in cases D and E is pathogenic, further analysis was conducted. *In silico* investigations by the predictive software indicated that the substitution of aspartic acid with glycine at codon 564 may be a disease-causing mutation. SIFT, Polyphen-2, MutationTaster, and FATHMM classified the p.A564G as a damaging variant. The conservation scores predicted by Phylop, PhastCons, and GERP++ were 4.32, 1, and 4.02, respectively. These analyses also revealed that aspartic acid 564 is located in the C-terminal domain of *KCNQ1* channel, in a highly conserved α-helix region. In addition, Mutation Mapper showed the aspartic acid at position 564 was conserved among 100 different species. Consequently, according to the American College of Medical Genetics and Genomics guidelines (ACMG), the recessive variant was categorized as a possible pathogenic mutation[29].

This is the first report of a novel homozygous frameshift mutation with JLNS. Based on ACMG, this mutation is likely to abolish channel function, severely. Identification of six JLNS and RWS index cases in a period of 24 months in a cardiogenetic clinic in Tehran suggests the high prevalence of LQTS. This high prevalence necessitates a broader surveillance in the country and reveals the importance of genetic and case series studies in precise detection of LQTS carriers’ frequency.
